# The Treatment of Traumatic Gangrene

**Published:** 1907-06

**Authors:** 


### THE TREATMENT OF TRAUMATIC GANGRENE.

Knott believes that when confronted with gangrene, in doing an amputation, two things should be avoided: first, the dissection of the flaps; second, the introduction of sutures. His method of procedure is as follows:

Being confronted with a case of traumatic gangrene of an extremity, estimate as exactly as possible the line between the diseased and healthy soft parts. Under anesthesia make a most careful and complete disinfection and cleansing of the skin, puncturing all bullæ

and remove all discharges, envelop the gangrenous area in a sterile towel up to the line selected and then, at this point, make a circular amputation, cutting through soft tissues and bone at the same level. Ligate carefully all bleeding points, including none of the perivascular tissue in the bite of either the forceps or the ligature. Leave the wound open, not introducing a single suture, and apply moist dressings of gauze saturated with salt solution, these dressings to be changed from two to four times in twenty-four hours, as the circumstances of the particular case demand.

After seven to ten days, if the wound is perfectly clean and the condition of the patient favorable, the classic circular amputation may be made by dissecting up the flap already outlined and sawing the bone at the proper level. If for any reason the circular method may seem undesirable, any other procedure may be substituted, but in the class of cases under discussion a typical circular amputation will be found entirely satisfactory.

It is unnecessary to report in detail the cases treated by this method, but it may be stated that since adopting it in 1900 I have employed it nine times: twice in amputations in the subtrochanteric area, twice in the middle third of the thigh, four times in the upper third of the leg, and once in the forearm. The results were eight recoveries and one death.—Jour. A. M. A., May 4, '07.

